# Cutting edge tools in the field of soil microbiology

**DOI:** 10.1016/j.crmicr.2024.100226

**Published:** 2024-02-21

**Authors:** Diksha Garg, Niketan Patel, Anamika Rawat, Alexandre Soares Rosado

**Affiliations:** a Department of Microbiology, Punjab Agricultural University, Ludhiana, Punjab, India; b Red Sea Research Center, Biological and Environmental Science and Engineering Division, King Abdullah University of Science and Technology, Thuwal, Makkah, 23955, Saudi Arabia; c Computational Bioscience Research Center, Biological and Environmental Science and Engineering Division, King Abdullah University of Science and Technology, Thuwal, Makkah, 23955, Saudi Arabia; d Center of Desert Agriculture, Biological and Environmental Science and Engineering Division, King Abdullah University of Science and Technology, Thuwal, Makkah, 23955, Saudi Arabia

**Keywords:** Soil microbiology, Metagenomics, Microarray, Microbiology, Next generation sequencing, NanoSIMS

## Abstract

The study of the whole of the genetic material contained within the microbial populations found in a certain environment is made possible by metagenomics. This technique enables a thorough knowledge of the variety, function, and interactions of microbial communities that are notoriously difficult to research. Due to the limitations of conventional techniques such as culturing and PCR-based methodologies, soil microbiology is a particularly challenging field. Metagenomics has emerged as an effective technique for overcoming these obstacles and shedding light on the dynamic nature of the microbial communities in soil. This review focuses on the principle of metagenomics techniques, their potential applications and limitations in soil microbial diversity analysis. The effectiveness of target-based metagenomics in determining the function of individual genes and microorganisms in soil ecosystems is also highlighted. Targeted metagenomics, including high-throughput sequencing and stable-isotope probing, is essential for studying microbial taxa and genes in complex ecosystems. Shotgun metagenomics may reveal the diversity of soil bacteria, composition, and function impacted by land use and soil management. Sanger, Next Generation Sequencing, Illumina, and Ion Torrent sequencing revolutionise soil microbiome research. Oxford Nanopore Technology (ONT) and Pacific Biosciences (PacBio)'s third and fourth generation sequencing systems revolutionise long-read technology. GeoChip, clone libraries, metagenomics, and metabarcoding help comprehend soil microbial communities. The article indicates that metagenomics may improve environmental management and agriculture despite existing limitations.Metagenomics has revolutionised soil microbiology research by revealing the complete diversity, function, and interactions of microorganisms in soil. Metagenomics is anticipated to continue defining the future of soil microbiology research despite some limitations, such as the difficulty of locating the appropriate sequencing method for specific genes.

## Highlights

• The use of metagenomics in soil microbiology is the subject of this review, which also discusses the potential and current limitations of the field.
• The several methods of metagenomic sequencing and how they are used in soil microbiology are discussed in this article.
• The advancements in metagenomics are anticipated to continue shaping the future of soil microbiology research, despite the limitations, such as the difficulty of finding the right sequencing method for specific genes.

## Graphical Abstract

**Figure:**
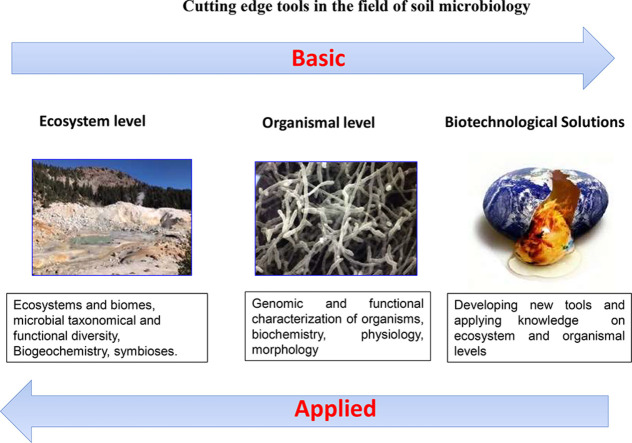

## 1 Introduction

Amongst the various complex ecosysthems on earth, soil is a crucial ecosysem essential for sustanence of life on earth (Raj *et al.*, 2019). Soil, a living entity on earth (Sabale *et al*., 2019), is a product of microbial as well as macrobial life, and has within it a diverse ecosystem – represented by a vast diversity and density of microbial communiites living in their own micro-environments in the soil. It is estimated that each gram of soil harbors more than 10^7^ of prokaryotic cells with a phylogenetic diversity ranging in thousands of microbial taxa (Gans *et al.*, 2005; Roesch *et al.*, 2007). The ability of soil to function as a living environment for humans, plants, animals, and microorganisms is known as soil health (Lehmann et al., 2020). However, some researchers disagree with this definition (Powlson, 2020). While agricultural productivity was the primary emphasis of soil assessments in the past, soil health now encompasses the function that soil plays in water quality, climate change, and human health. Nevertheless, despite rising recognition of the significance of soil biodiversity, chemical markers continue to dominate the quantification of soil health due to a lack of useful techniques and functional knowledge. This concept is based on the assumption that soils are multipurpose living systems sustaining diverse soil biota and are essential to the structure and function of both natural and agricultural systems (Pulleman et al., 2022).

The soil microbiome is crucial for the sustainability of life on earth as these microbial communities ensures healthy plant growth, regulates soil health and are indispensable for proper functioning of important biogeochemical cycles, thereby maintainting the proper recycling of organic and inorganic matters in the terrestrial environemtn. Therefore, researchers have focused on environmental remedies and agricultural methods that promote soil diversity and soil quality-enhancing microbes to ensure long-term food security (Rachid et al., 2023). Although the soil microbiome constitutes an enormous number of microbes, only a fraction of those microbes (0.1 - 51%) are cultivable in lab conditions, necessitating a need for new and sophiscated technologies to study microbiome as a whole (Torsvik *et al.*, 1990; Torsvik & Øvreås, 2002; Mocali & Benedetti, 2010; Chaudhary *et al.*, 2019). These “uncultivable” microorganisms are unexplored reservoirs of species with unique biological and chemical characteristics. In their natural habitat, uncultivable bacteria require energy, but they cannot grow in a laboratory medium (Mu et al., 2021) creating a gap in knowledge about these species’ habitats, organic and inorganic interactions, and biological roles in soil (Gwenzi, 2021). The term “ uncultivable ” is a misnomer because it implies that these species can never be cultivated. Several researchers are currently studying uncultivable microorganisms and developing methods to do so. However, more than 99% of soil bacteria are yet to be cultivated. The presence of functional traits in such uncultured bacteria has been demonstrated repeatedly using molecular methods (Romillac and Santorufo, 2021). It is important to investigate the biotechnological applications and untapped abilities of these uncultivated microorganisms to produce bioactive molecules and hence it gets necessary to isolate uncultured microorganisms in the laboratory to accomplish these objectives (Sedeek et al., 2022). Unfortunately, research in this field is still in its early stages. Failure to establish natural growing conditions is the primary barrier to bacterial growth in laboratory medium, and certain bacteria are unculturable because of a lack of knowledge about specific parameters, including multiplication time, suitable temperature, and nutritional requirements for growth (Chaudhary et al., 2019). The utilization of nutrient-rich medium and the creation of an environment that supports fast-growing species are the main goals of conventional methods for cultivating bacteria. However, using certain type of culture media and growth condition which is optimal for some microbes may be detrimental to other categories of microbial species, for eg. nutrient-rich environments can be harmful to bacteria that thrive in nutrient-poor environments. It has been demonstrated that traditional approaches favor fast-growing bacteria and undervalue slow-growing bacteria. Agar is a solidifier that is commonly used in culture medium, which sometimes can prevent the reproduction of bacteria. On the contrary, gellan gum has been demonstrated to accelerate the growth rates of a variety of microorganisms when used instead of agar (Gupta et al., 2022). Additionally, for certain organisms, specific indicator agents such as ligands are required to determine a favorable environment in which they can grow. Furthermore, some microbes can grow in the presence of assistant chemicals and helper microorganisms. Because microorganisms need iron for growth, chemicals such as siderophores can be added to solubilize iron such that it is accessible to microorganisms (Miller et al., 2021). Similarly, by reducing oxidative stress, certain helper microbes can protect other species from the damaging effects of the environment (White et al., 2019). Helper-dependent bacteria cannot conduct crucial metabolic pathways without the support of environmental assistant agents and helper organisms (Sommers et al., 2021).

The interaction of bacterial diversity and physiology is crucial for their growth and is influenced by several factors and variables, including temperature, lack of nutrition, osmotic stress, pH, and other elements. These must be addressed in order to replicate natural conditions for the growth of microorganisms (Hug et al., 2020). However, given the enormous importance of soil microbes for all major aspects of biological systems, researchers are always in need of novel methods to access the huge amount of information hidden within the whole genome of soil microbiome. This “collective DNA of all the indigenous soil biota” is referred to as *metagenome* (Handelsman et al., 1998) and the approach to study the metagenome isolated from all the organism, by bypassing the need for isolation and growth under laboratory condition, is called *“Metagenomics”*. In recent years, researchers have attempted to culture various uncultivated microbial species after metagenomics investigations uncovered uncultivated microbial phylogenetic clades. Uncultivable bacteria have been successfully grown using a variety of techniques, including co-culturing with helpers, mimicking natural environments, extending incubation time, and changing the nutritional composition of the media and growth conditions. Recent advancements in culture techniques include optical tweezers, transwell plates, microbioreactors, diffusion chambers, and laser microdissection (Chaudhary et al., 2019).

Second- and third-generation sequencing techniques, which can provide hundreds of gigabytes of DNA sequence data at a low cost, have fueled improvements in metagenomics (Akaçin et al., 2022, Bansal et al., 2018). Because of these advances in sequencing depth, it is now possible to represent even the most uncommon microorganisms in an ecosystem. Owing to the advancements in sequencing technologies and bioinformatics, metagenomic analysis is now a practical, rapid, and economical tool for most laboratories (Maljkovic et al., 2020). Although most studies on the microbiome focus on bacteria and archaea, the term “microbiome” refers to the entire population of microorganisms living in any particular environment. Marker gene–based experiments and whole genome shotgun metagenomics are the two major approaches in high-throughput microbiome research. In marker-gene research, each genome present in a sample, for example, 16S rRNA for bacteria and archaea or 18S rRNA for fungi, is amplified using polymerase chain reaction (PCR) (Tkacz et al., 2018). The product is then sequenced. Operational-taxonomic units are created from the sequences, which are then compared among samples (Wei et al., 2021). The microbiome may produce a variety of novel enzymes and biocatalysts with significant commercial uses, including in the biofuels, biotechnology, and pharmaceutical industries (Fasim et al., 2021). In extensive metagenomic research, in addition to detecting approximately 27,000 potential carbohydrate-active enzymes (CAZy) exhibiting cellulolytic activity, Hess et al. (2011) discovered over 2.5 million distinct genes. They also discovered 15 microbes with almost completely unexplored genomes (Lopes et al., 2015). Over 1.5 million potential genes were discovered by annotating the resulting contigs, with unknown protein domains in 58% of them (Stewart et al., 2019). As an in-depth examination of the genetic diversity inside various microbial communities, metagenomics is essential for thorough study of antibiotic resistance. Through the use of this methodology, the intricate relationship between functional genes linked to antibiotic resistance mechanisms may be defined offering an improved comprehension of their characteristics and roles in microbial functions. The use of metagenomics has significant implications for the advancement of environmental research and biotechnology. The use of functional metagenomics to examine copper (Cu) resistance genes in soil metagenomic libraries is an example to demonstrate effectiveness for studying metal resistance genes. The investigation done by Xing et al., (2020) found 12 distinct sequences including 25 probable open reading frames linked to the ion transport/chelation, signal transduction, membrane components, energy metabolism, and Cu stress response pathways. A bioinformatics research revealed their participation in several biological functions. The diverse biomass and Cu sorption capabilities shown by the re-transformed clones highlight the unexplored variety of microbial Cu resistance genetic determinants in soil ecosystems and suggest that these Cu resistance genes may play a role in the development of innovative bioremediation techniques. Since it makes it possible to thoroughly analyse antibiotic resistance genes (ARGs) in benign environments, metagenomics is essential to our knowledge of antibiotic resistance. In the research conducted by Yuan et al., (2019), which concentrated on comparatively unspoiled Antarctic soils, metagenomic techniques identified 79 ARG subtypes connected to 12 kinds of antibiotics, with the predominance of ARGs linked to polypeptide and multidrug resistance. The unique makeup, quantity, and possible transferability of ARGs in Antarctic soils relative to other settings highlight the importance of metagenomics in elucidating the dynamics and evolution of antibiotic resistance, offering insightful information for environmental and therapeutic concerns.

The varied and intricate nature of microbial communities in soil ecosystems is one of the main problems and obstacles that soil microbiology research must overcome. The whole range of microbial diversity cannot be fully captured by traditional cultivation-based approaches since many soil microorganisms are challenging to cultivate in a lab. Tools for metagenomics have become effective means of addressing these issues. The identification and characterisation of unculturable microorganisms, which make up a significant fraction of soil microbial populations, is one important problem. By directly analysing the collective genetic material from environmental samples, researchers may get insights into the functional potential of the whole microbial community via the use of metagenomics. Understanding the complex relationships that soil bacteria have with their surroundings, such as the cycling of nutrients, interactions between plants and microbes, and reactions to environmental stress, is another difficulty. Through the investigation of microbial community dynamics, soil ecosystems' intricate web of interactions may be better understood by using metagenomics. Furthermore, there is considerable concern about the way human activity affects the microbial ecosystems in soil. The development of sustainable soil management techniques is aided by the evaluation of the impacts of pollution, climate change, and changes in land use on soil microbiota via the use of metagenomic methodologies. Because metagenomics technologies provide a comprehensive and high-throughput investigation of microbial communities in their natural environment, they are crucial in furthering our knowledge of soil microbiology.

Without prior knowledge of microbial communities, microbial genomes can be collected directly from environmental samples for environmental genomics, community genomics, and metagenomics (Cordier et al., 2021). The term "metagenomics" refers to the practise of sequencing the whole genomes of isolated microbial communities, just like one would read the complete genome of a single bacterium. Thus, it may be used to learn about the metabolic processes of microorganisms and how they respond to a variety of environmental and biotic factors (Kunda et al., 2018). Whole genome metagenomics is useful for assessing the functional gene capacities of the microbial community (Praeg et al., 2020). Soil metagenomics relies on soil DNA extraction, followed by clone library construction and screening. The discovery of novel genetic biomolecules has been significantly impacted by the identification, appraisal, and cultivation of large pools of varied genetic material among soil microorganisms (Nural et al., 2022). However, scientists agree that the vast bulk of the world's microbes have yet to be cultured. An important method for understanding the function of archaea is metagenomics, as shown by the investigation of Methanomassiliicoccales (Borrel et al., 2017). Through the examination of operational taxonomic units, global distribution patterns, and functional genomics, metagenomics provides light on the dynamic interactions and possible consequences of these archaea in complex soil microbial communities. This method provides a more detailed knowledge of the functional capabilities, diversity, and adaptation strategies of archaea in certain ecological niches. In order to address the issues raised by the "uncultivated majority" of bacteria, the research by Genomic Standards Consortium (GSC) has presented relies heavily on metagenomics. Without the necessity for culture, metagenomic techniques like single-cell genomics and shotgun metagenomics are crucial in revealing the genetic diversity of bacterial and archaeal communities. The research conducted by Bowers et al., (2017) highlights the significance of establishing guidelines for the production, depositing, and disseminating genomes originating from uncultivated bacteria and archaea. Two fundamental guidelines created by the GSC—the Minimum Information about a Single Amplified Genome (MISAG) and the Minimum Information about a Metagenome-Assembled Genome (MIMAG)—are made easier to apply by metagenomics. These guidelines allow for the thorough reporting of genomic data, including contamination, estimations of genome completeness, and assembly quality. They work in conjunction with the already-existing GSC criteria. Adopting these metagenomic standards throughout the community is essential to promoting strong comparative genomic studies and improving our knowledge of the variety of bacteria and archaea in intricate microbial ecosystems. The abundance, taxonomic diversity, and ecological interactions between DPANN (Diapherotrites, Parvarchaeota, Aenigmarchaeota, Nanohaloarchaeota, and Nanoarchaeota) archaea and Candidate Phyla Radiation (CPR) bacteria in groundwater habitats are mostly revealed by metagenomics (He et al., 2021). The study found 746 CPR and DPANN genomes using genome-resolved metagenomic analysis, revealing host relationships and site-specific differences in their distribution. Together with genomic analysis and cryogenic transmission electron microscopy, metagenomic findings provided light on adherence of CPR and DPANN lineages to their host cells and raise the possibility that host-cell surfaces may promote the proliferation of CPR bacteria. The site-specific diversity of these microbial communities in groundwater is better understood with the help of metagenomic approach.

Shotgun sequencing techniques require libraries along with Illumina sequencing, generates more sequences with a saturated genome size of approximately 4.0 Gbps (Biswas & Sarkar 2018) the output of shotgun sequencing when coupled with Illumina platform is upto 1.5Tb per run (https://www.cd-genomics.com/introduction-to-shotgun-metagenomics-from-sampling-to-data-analysis.html). Individual readings and the construction of contigs both increase the functional potential of screening the soil microbiota's energy metabolism. It also aids in distinguishing various microorganisms in the environment, which are both complex and diverse. Other functional compounds, such as antibiotics (e.g., terragine) and microbial enzymes (e.g., amylases, lipases, and cellulases), may be produced from functional metagenomic libraries (Dhanjal et al., 2018). Therefore, to accurately characterize the microbiological sample and its equivalents, enhanced detection techniques and high-throughput screening are required. This literature review presents the most advanced and credible research on the use of various technologies in soil microbiome studies. High-throughput sequencing (HTS) is a method for characterising the network of soil microbiomes. NGS technology is rigorously reviewed to offer some improvements. Some software applications for data processing of metagenomic sequences from the microbiome are also studied.

This comprehensive review emphasises the value of targeted metagenomics in elucidating certain microbial taxa and genes in complex ecosystems. Direct research of genetic diversity are made possible by methods like as high-throughput sequencing and stable-isotope probing, which also provide light on environmental variables, microbial ecology, and potential uses in bioremediation and disease prevention. An effective method for examining the diversity, composition, and function of soil bacteria that are influenced by soil management and land use is shotgun metagenomics. Sanger and Next Generation Sequencing (NGS) are two examples of high-throughput sequencing technologies that are revolutionising soil microbiome research by allowing in-depth investigation of microbial populations. The paper explores the sequencing techniques of Illumina and Ion Torrent, highlighting their applications in soil microbiological research. Platforms for third and fourth generation sequencing, such those offered by Oxford Nanopore Technology (ONT) and Pacific Biosciences (PacBio), represent a radical move towards long-read technology. Complementary methods such as GeoChip, clone libraries, metagenomics, and metabarcoding aid in the comprehension of soil microbial populations. In spite of current obstacles, the paper concludes by highlighting metagenomics' potential to improve environmental management and agriculture.

## 2 Targeted Metagenomics

Targeted metagenomics is an effective method that allows scientists to study specific groups of microorganisms or genes within complex microbial communities. This method is used to investigate the functional roles of microorganisms in a specific environment and to understand how they interact with each other and with their environment. In targeted metagenomics, Extraction of DNA from a material (such as soil or water) is followed by targeted amplifications of that DNA in order to isolate or study certain microbes or genes of interest. For example, the DNA can be amplified using PCR primers that target a specific group of microorganisms or genes, such as those involved in a specific metabolic pathway. Alternatively, the DNA can be enriched using baits, such as ribosomal RNA probes, that capture specific groups of microorganisms based on their genetic similarity to known organisms. Once the targeted DNA has been amplified or enriched, it can be sequenced using methods of high-throughput sequencing, such as next-generation sequencing (NGS), to generate large amounts of data. The data can then be analyzed to determine the abundance and diversity of the targeted microorganisms or genes, and to identify their functions and interactions with other organisms and the environment. Targeted metagenomics is a useful tool for investigating the ecology and evolution of microorganisms in a diverse environments, including soil, water, and the human gut. It has also been used to study the role of microorganisms in important processes, such as bioremediation, and to develop new strategies for controlling the spread of diseases and managing environmental problems.The field of microbial ecology benefits greatly from targeted metagenomics. It helps scientists decipher how many environmental factors are connected to the structure and function of genes. Furthermore, it allows for the direct investigation of the vast genetic variety of microbial communities, even when more than 99% of microorganisms are not capable of being cultured (Suenaga 2012). Targeted metagenomics allows researchers to get a greater knowledge of the functional, ecological, and evolutionary patterns of key genes in microorganisms from diverse habitats by choosing particular subsets of metagenomes for sequence analysis. Because of the difficulty in getting sufficient sequencing depth of any one gene to accurately capture the complexity of microbial metagenomes and make meaningful conclusions about microbial communities, researchers have turned to the targeted metagenomics technique. Targeted metagenomics is a method that combines stable-isotope probing (SIP) with metagenomics to explore the microbial diversity and function in a specific environment. This approach enables the recovery of genomes from microorganisms involved in metabolic processes of interest by incorporating stable isotopes into microbial biomass (Coyotzi et al., 2016). The resulting labeled nucleic acids can then be analyzed using metagenomic analysis to identify novel enzymes for biotechnology applications. Targeted metagenomics is an effective technique for applied and environmental microbiology, and its use is expected to expand in the future as it continues to provide valuable insights into the metabolic processes of microorganisms in various ecosystems.

It helps scientists decipher how many environmental factors are connected to the structure and function of genes of soil microbial flora. Furthermore, it allows for the direct investigation of the vast genetic variety of microbial communities, even when more than 99% of microorganisms are not capable of being cultured (Suenaga 2012). Targeted metagenomics examined methanotroph diversity, activity, and metabolism in the Tibetan Plateau Zoige wetland, a major methane emitter (Yun et al., 2021). Methane-oxidizing bacteria reduce its environmental impact. DNA stable isotope probing (SIP) with 13C-labeled methane examined Zoige wetland methanotrophs. Active methanotrophs incorporated 13C-labeled methane into DNA. Active methanotrophs and their metabolic pathways may be detected by sequencing labelled DNA. Labeled DNA found active methanotroph metagenome-assembled genomes (MAGs). MAGs are created using ambient metagenomic data without microbial cultivation. MAGs showed two distantly related methanotrophs. Gammaproteobacteria type I methanotrophs MAGs have mxaF and xoxF methanol dehydrogenase genes. H4MPT and RuMP oxidised methane in these methanotrophs. The Zoige wetland's gammaproteobacteria oxidised methane. DNA-SIP targeted metagenomics discovered active methanotrophs in the Zoige wetland and characterised their metabolic pathways. The finding of novel methanotroph MAGs highlighted the need for metagenomics to study environmental microbial populations, which may reduce methane emissions and ameliorate global warming.

Targeted metagenomics discovered *Saccharomyces* species in Italian Alps soils around trees (Alsammar et al., 2019). Culture limitations may invalidate *Saccharomyces* yeasts in soil and bark. Researchers isolated ITS region of *Saccharomyces* to detect yeast species. Targeted metagenomics amplifies and sequences chosen DNA. All selected soil samples had *Saccharomyces mikatae* and *S. eubayanus*. The ITS1 sequences of *S. cerevisiae, S. paradoxus*, and *S. kudriavzevii* were compared to other strains and discovered up to three base pair polymorphisms, suggesting new lineages. *Saccharomyces* species were found in their original habitat during selection, even though basidiomycetous fungus outnumbered Ascomycota. Targeted metagenomics amplified and sequenced ITS region of *Saccharomyces*, demonstrating its richness. Findings are needed to understand *Saccharomyces* species distribution and abundance in nature and microbes.

Gene-targeted metagenomics assessed PAH-degrading bacterial diversity in oilfield soils and mangrove sediments (Liang et al., 2019). PAH-degrading bacteria biomarker PAH-RHDα was examined. PCR and cloning biases hide microbial community diversity in clone library analyses. PAH-RHDα gene-targeted metagenomics. Gene-targeted metagenomics discovered more PAH-degrading bacteria than clone libraries. *Pseudomonas, Burkholderia, Ralstonia, Polymorphum gilvum, Mycobacterium, Sciscionella marina, Rhodococcus*, and perhaps novel degraders were the oilfield's leading PAH-degraders. *Mycobacterium* and new PAH degraders dominated mangrove sediments. PAH-RHDα gene distribution depends on local environmental conditions. PAH boosted the PAH-degrading bacterial population and diminished its diversity. Gene-targeted metagenomics may uncover soil and sediment PAH-degrading bacteria, improving microbial community investigations.

So, targeted metagenomics approach enabled the recovery of genomes from microorganisms involved in metabolic processes of interest by incorporating stable isotopes into microbial biomass (Coyotzi et al., 2016). The resulting labeled nucleic acids can then be analyzed using metagenomic analysis to identify novel enzymes for biotechnology applications.

Targeted metagenomics is crucial to studying aquatic microbial bacteria ecology and evolution. Aquatic microbial communities are diverse and vital to food web dynamics and biogeochemical processes. Metagenomic methods have quickly emerged to link microbial community dynamics to ecosystem-scale biogeochemical changes. Metagenomic methods and recent findings, including new taxa and metabolisms, community assembly and functional ecology, evolutionary processes shaping microbial genomes and microbiomes, and human impacted on aquatic microbiomes have been discussed. Since metagenomics was used in well-designed ecological experiments, informing and validating metabolic and biogeochemical models, and the importance of ecologically relevant model organisms and simple microbial systems for interpreting taxonomic and functional information in metagenomes. These research areas aimed to improve mechanistic and predictive knowledge of the complex relationships between microbial dynamics and biogeochemical cycles, which is critical given fast climate change and human influences on aquatic ecosystems.

Targeted metagenomics revealed community dynamics, biogeochemical processes, and evolutionary impacts in aquatic microbial microorganisms. This advance technology helped toidentify novel species, metabolic pathways, and human impacts on aquatic microbiomes. Metagenomics in ecological experiments, model validation, and ecologically relevant model species for mechanistic understanding of microbial dynamics amidst climate change and human influences are research areas (Grossart et al., 2020). Targeted metagenomics helped understand amoeba-bacterium ecology and evolution, including predation, symbiosis, and disease. The varied links between amoebae and bacteria, ecological interactions, and their results are reviewed by Shi et al., (2021). These interactions affect human health, horizontal gene transfer, drinking water safety, and symbiosis evolution. Future studies should use advanced metagenomics approaches to fill research gaps and better comprehend hidden diversity, amoeba genomes, and microbiome predation. In palaeomicrobiology, targeted metagenomics using Next Generation Sequencing (NGS) is crucial to studying microbe ecology and evolution. NGS has revolutionised the study of ancient non-cultivable bacteria, allowing genome reconstruction, pathogenicity research, and comprehensive microbial evolution. Beyond infections, focused metagenomics in palaeomicrobiology may examine commensal bacteria, non-human host species, behaviour, migration, and culture. Study conducted by Arriola et al., (2020) provides a unique way to study bacterial evolutionary history on a larger scale, highlighting its promise for non-pathogenic microbe research. Targeted metagenomics is essential for understanding soil bacteria-associated viral ecology and evolution. A research using metagenomic data from northern California grassland soil found 10,196 non-redundant viral operational taxonomic units (vOTUs) at various soil thicknesses (Muscatt et al., 2022). Lysogeny did not dominate soil viral communities, contrary to previous theories. The research revealed complex antagonistic co-evolution in surface and subsurface soils by studying viral diversity at macro and micro levels. The research also showed soil viruses remineralizing soil carbon. Targeted metagenomics is important for studying viral ecology and its effects on soil microbial interactions and biogeochemical processes. The results highlight the necessity to study subsurface viral ecosystems to better understand soil viral ecology.

Targeted metagenomics helped understand soil microbial community ecology and evolution, including viral contributions to terrestrial ecosystems. This microcosmic research by Santos-Medellín et al., (2023) uses simulated precipitation to depict short-term dynamics during seasonal drought in Mediterranean grasslands. Through viromes, metagenomes, and 16S ribosomal RNA gene amplicon datasets, the study shows diverse viral populations following similar successional paths during wet-up episodes. Targeted metagenomics showed viral richness, compositional change, and virus predation of dominant bacterial species rise rapidly. The research showed that focused metagenomics was conserved in studying microbial mortality, nutrient cycling, and soil trophic network connections. Targeted metagenomics was essential for understanding termite digestive system microbial ecology and evolution. The study (Moreira et al., 2021) used ITS sequence analysis, metagenomics, and metatranscriptomics to examine the differential expression of genes encoding carbohydrate-active enzymes (CAZy) in the gut and specialised food nodules in *Cornitermes cumulans* during lignocellulose digestion. Food nodules express a different set of CAZy genes, indicating an externalised digesting process targeting lignin and complex polysaccharides before entering the termite stomach. This study showed how focused metagenomics may reveal the collaborative interactions between gut symbionts, host enzymes, and exterior nest bacteria, revealing termite evolution's complementing digestive mechanisms.In conclusion, targeted metagenomics is a powerful tool for investigating complex microbial populations and understanding the roles and interactions of microorganisms in specific environments. The approach provides a means for studying microorganisms at a deeper level of detail than is possible with traditional culture-based methods, and is helping to advance our understanding of the complex relationships between microorganisms and their environments. Targeted metagenomics is an effective technique for applied and environmental microbiology, and its use is expected to expand in the future as it continues to provide valuable insights into the metabolic processes of microorganisms in various ecosystems. In conclusion, targeted metagenomics is a powerful tool for investigating complex microbial populations and understanding the roles and interactions of microorganisms in specific environments. The approach provides a means for studying microorganisms at a deeper level of detail than is possible with traditional culture-based methods, and is helping to advance our understanding of the complex relationships between microorganisms and their environments. One of the most effective genomic approaches is shotgun sequencing, which produces DNA reads that are usually between 400 and 500 base pairs long. When the genetic information is available, this method works very well for identifying the species or strain of an organism. Researchers may examine millions of reads from next-generation sequencing of environmental samples using taxonomy classifier software, providing a thorough understanding of complicated microbiomes, such those found in the gut flora. One significant benefit of shotgun sequencing over 16S rRNA amplicon sequencing is that it is not confined to bacteria. Shotgun metagenomics reveals soil bacteria's composition, diversity, and function. It may reveal soil microbial nutrition cycle and carbon sequestration. Shotgun metagenomics examined how land use and soil management affect soil microbes. Furthermore, strain-level classification is possible with shotgun sequencing, which is more accurate than genus-level classification with amplicon sequencing. Additionally, it makes it possible to isolate whole genes, giving researchers the ability to identify their roles within the metagenome. Notwithstanding, it is important to recognise and confront obstacles associated with potential contamination of the sample or sequencing pipeline, underscoring the need of stringent quality control protocols in the procedure. Shotgun metagenomics may reveal soil's richness in bacteria and fungus, cultureable and not. This may help microbial culture and research. Shotgun metagenomics is needed to study soil microbes and their effects. Amplicon sequencing misses over 90% of shotgun metagenome KS and A domains that create polyketides (PKS) and nonribosomal peptides (NRPS) (Mantri et al., 2021). Shotgun and amplicon metagenome sequencing discover domains. 1,571 of the 181,324 A domain amplicons and 638 of the KS amplicon-seq amplicons did not match any KS shotgun-seq OBUs.

A measure of biodiversity known as alpha-diversity evaluates the richness or variety of species found in a particular environment or community. It measures the diversity of species found in a particular ecosystem or place, providing information on the richness and evenness of the local species (Walters et al., 2020). Species richness, or the quantity of distinct species, and Shannon's diversity index, which considers both species richness and evenness of abundance, are common measures for alpha-diversity. In ecological research, alpha-diversity is often used to compare biodiversity across various habitats or monitor changes in diversity over time within a single habitat. It offers a snapshot of the richness at a given location. Alpha diversity indicated biochemical domain and microbial community trends. Shotgun-seq and amplicon-seq diversity were unrelated. All Kraken 2 shotgun metagenomes contain 52.95% unidentified and 47.04% classified reads (Wagner et al., 2018). Actinobacteria and Proteobacteria dominate metagenome annotations. BiG-MEx finds BGCs and analyses diversity in unassembled metagenomes. BiG-MEx annotated 150 A-type BGC domains (Rego et al., 2021). Shotgun metagenome sequencing identified >90% A and KS domains. Shotgun metagenome and amplicon sequencing identified domains. Quick shotgun data collection shows its pros and cons. A metaSPA-based hybrid Illumina-cambisol nanopore assembly was created to study BGC recovery and nanopore read durations. Hybrid assembly considerably lengthened contigs. Illumina contigs were seven times shorter than hybrids. 598,670 bases. Amplicon and shotgun metagenome sequencing of soil samples showed product zones and BGCs. Shotgun-seq and 16S amplicon-seq demonstrated microbial composition alterations. Proteobacteria and Actinobacteria dominated shotgun-seq, whereas Planctomycetes dominated amplicon-seq. Amplicon-based shotgun metagenome sequencing overestimated alpha diversity. Shotgun-seq datasets may provide full-length BGCs with higher-depth sequencing and biosynthetic domain diversity. Biochemical diversity trend mining software requires massive data sets.

Amplicon-based microbial community investigations extract DNA from soil, dung, charcoal, compost, or consortiums. PCR amplifies fungal, yeast, and oomycete markers. 18S or ITS sequences eukaryotes (protists) (De Corato, 2020). Amplicon sequencing examines microbiome bacteria. Bioinformatics organises and annotates sequencing data for insight. Amplified sequencing monitors taxonomic microbial population changes. 16S rRNA sequencing distinguishes bacteria. Metabarcoding is cheaper than shotgun sequencing for sample taxonomy. Shotgun metagenomics sequences strains and microbiomes (Czech et al., 2022). It assessed habitat genes and population diversity in situ (Wambugu and Henry, 2022).

The 16S rRNA gene sequences most bacterial genera but cannot predict their species. Common 16S rRNA gene activity prediction technique PICRUSt1 is incompatible with sequence denoising methods that create amplicon sequence variances (ASVs). ASVs recognise closely related species better than OTUs. New genome prediction technique PICRUSt2 improves functional predictions (Douglas et al., 2020). Genome prediction improves with a reference phylogeny and a bigger library of reference genomes and gene families. Open-source approaches infer ambient 16S rRNA gene genomes and add ASVs to a reference tree for functional predictions in PICRUSt2. This technique improves taxonomic diversity over PICRUSt1.

Joos et al. (2020) compared Belgian field soil ASV, usASV, and OTU bacterial population analysis methods. While minor, unfiltered ASV generated fewer ASVs than OTU. Technical filtering reduced ASVs by 70% and OTUs by 15%. ASV decreased soil dataset community diversity more than OTU and usASV. ASVs are five times worse than OTUs and twice worse than usASVs. ASV richness reached 12,000 sequences, although OTU richness increased beyond 125,000. Unlike OTUs, ASVs deepened sequencing. All techniques yielded Shannon variety and richness.

NovaSeq Illumina outperforms HiSeq. NovaSeq sequences 6 Tb each run, double HiSeq X's 3.5 Tb (Zhou et al., 2019). NovaSeq's flow chambers boost throughput. NovaSeq wins. NovaSeq S4 flow cells produce 500 Gb in two days, whereas HiSeq X takes three. NovaSeq flow cells improve variant calling and genome coverage. Researchers choose NovaSeq flow cell architecture. NovaSeq S1 flow cells do low-coverage whole-genome sequencing and S4 exome sequencing. NovaSeq costs more per Gb than HiSeq X, but experimental methods and sequencing chemicals will improve. NovaSeq's throughput and data quality may minimise downstream operating costs, making it a superior long-term investment for many research projects. Metagenomics is crucial since it reveals Yumesamdong hot springs riverine bacterial diversity. Metagenomics explores over 2000 bacterial and archaeal species biodiversity and major phylum fluctuations throughout heat gradients using culture-dependent and culture-independent methods, including high-throughput sequencing (Kumar et al., 2023). The antibiotic resistance patterns connected with temperature gradients illustrate that metagenomic techniques may link microbial community dynamics to abiotic variables, notably temperature, and reveal complex ecological and evolutionary patterns. The research conducted by Das et al., (2023) relies on metagenomics to build and characterise metagenome assembled genomes (MAGs) from Indian Himalayan Geothermal Belt hot springs. Metagenomics classified thermophilic and mesophilic bacteria, including archaeal genomes, using databases and tools. Functional characterization, particularly the identification of carbohydrate-active enzymes (CAZymes), antibiotic resistance genes, and heavy metal tolerance genes, showed the potential of metagenomic approaches to reveal geothermal ecological and functional potential of microbial communities.

Microbiome studies enhanced fungal ITS1. Amplicon HTS's high sequencing output led to first- and second-generation integrated systems (Roche 454-pyrosequencing and Illumina/Solexa). PacBio, Ion Torrent, and Oxford Nanopore third- and fourth-generation HTS analyse soil microbiota better (Nilsson et al., 2019). The NCBI UNITE database can identify soil fungus sequence-based taxonomy and phylogenetic relationships using the most accurate and consistent bioinformatics approaches (De Corato, 2020). Modern metagenomics libraries and databases include disease-fighting microbial communities. The Sequence Read Archive (SRA) database stores researchers' microbiome sequencing data as a new bioproject and provides each sequence an international identification code number. SRA offers precise microbiota structure to uncover functional genes and maybe unrecognised BCAs that impact plant diseases throughout microbiome changes, including abundance, uniqueness, variety, symmetry, and composition. Many methods analyse amplicon sequencing data. EMP and GSC soil microbiome research standards simplify bioproject meta-analyses. EMP and GSC aid soil microbiome researchers and standardise bioinformatics procedures.

## 3 High throughput sequencing technology

### 3.1 Next Generation Sequencing (NGS)

Next Generation Sequencing (NGS) is a revolutionary technology in biological sciences which has enabed researchers to study genomes and transcriptomes at ease due to its ultra high throughput, high sensitivity, speed, genome coverage and cost effectiveness when compared to traditional Sanger Sequencing (Table 1). The NGS is often classified based on the read length as: short read/second generation technologies and long read/third generation technologies (Knief, 2014). Unlike Sanger sequencing, the NGS technology is based on construction of DNA library to which the synthetic DNAs (adapters), which are specific to each sequencing platform, are covalently added to each DNA fragments to be sequenced. These library fragments are amplified *in situ* where millions of reactions occurs in parallel and generate enormous data sets. Thus NGS platforms allow multiple sequencing reactions and detection at the same time (Shendure & Ji, 2008). However, along with the short read length (100 to 400 bp) one of the another drawback of the second generation sequencing is that it is a lengthy sequencing method, and thus real-time sequencing data cannot be attain from such platforms (Gehrig *et al.*, 2022). To overcome the drawbacks of second generation sequencing, and due to the techonological advancement in the sequencing field, third generation sequeincing or single molecule sequinging technology, was developed. The third generation technology can give subtantially longer reads (over 10,000bp) (Cao *et al.*, 2017; Gunjal *et al.*, 2023) and unlike second generation sequencing which relies on library construction, it can perform sequencing direclty on original DNA/RNA samples reading upto 10 nucleic acids per second thus significantly reducing the sequencing time (Vasudeva *et al.*, 2023). The third gen sequeincing can also be used for real time data accusation due to its capability of generating ultralong read lengths wich can be used to generate full length mRNA sequences (Metzker, 2010).

**Table 1:** Table enlisting applications of NGS in soil microbiology, along with their advantages, disadvantages, operative conditions, and important factors for NGS

Application	Operative conditions	Important factors for NGS	Advantages	Disadvantages	References
Analysis of Microbial Diversity	Taking samples and removing DNA from soil samples PCR amplification and indexing are examples of library preparation. NGS systems (like Illumina and Ion Torrent) for sequencing	DNA extraction and library preparation quality assurance. The optimisation of read length and sequencing depth Tools and workflows for data analysis in bioinformatics	Enables thorough evaluation of the variety of microbes in soil.	NGS technologies have the potential to produce enormous volumes of data, which requires computational to analyse.	Conrads and Abdelbary (2019)
	Provides taxonomic identification with great resolution.	Probability of biases and sequencing mistakes affecting the accuracy of the data.			
	Allows identification of uncommon and inaccessible bacteria.	Budgetary issues, since NGS might be more costly than conventional techniques.			
Functional Metagenomics	Gathering samples and removing DNA from soil samples.- The creation of metagenomic libraries.	Choosing the right sequencing depth to ensure functional coverage.- Stable bioinformatics pipelines for route analysis and functional annotation	Enables the identification of useful genes and biochemical pathways in soil microbiomes.	Complex bioinformatics processes and data analysis are needed to understand functional metagenomic data.	Taş et al., (2021)
			Makes it possible to find new enzymes and metabolic capacities.	Difficulties in tying individual microbes in complicated soil communities to functional genes.	
Microbial Community Dynamics	Extracting DNA/RNA from soil samples- NGS platforms for sequencing (DNA or RNA sequencing)-	Environmental parameter and condition metadata collecting- Statistical methods for studying community dynamics- Integration of datasets from multi-omics	Offers temporal and geographic insights on changes in microbial communities.	Limited capacity to distinguish between dormant and active bacteria.- Sample collection at various sites or times.-	Kumar et al., (2021)
			Enables the monitoring of community reactions to environmental changes.	Difficulties in using DNA/RNA sequencing alone to determine functional activity.	
Gene Expression Analysis	Sample collection at chosen intervals or circumstances- The removal of RNA from soil samples- NGS platform sequencing- samples-	Improving RNA extraction techniques for soil Library preparation methods appropriate for RNA-seq- Reliable normalisation and techniques for analysing differential expression.	Makes it possible to measure the levels of gene expression in soil microbial communitie Library building, such as creating an RNA-seq library.-	Extraction of RNA from soil samples may be difficult and subject to bias.-	Segawa et al., (2021)

Next-generation sequencing assessed insecticide-induced soil bacterial activity (Romdhane et al., 2019). Next-generation sequencing examined soil bacterial community diversity and structure using 16S rRNA gene amplicons. Leptospermone and sulcotrione were tested on non-target soil bacteria. Soil metabolomics and next-generation sequencing followed herbicides and their transformation products in soil microcosms. This approach revealed β-triketone herbicide ecotoxicity biomarkers in non-target soil microbes. Incubation eliminated leptospermone and sulcotrione, although transformation products of natural and synthesised β-triketone were found.

Next-generation sequencing examined the mycobiome of long-term crude oil-contaminated soils (Gałązka et al., 2020). Sequenced fungal DNA ITS. Biolog FFPlates assessed fungal community functional diversity using sequencing data. To evaluate the fungal community's bioremediation potential, the study measured glomalin related soil protein (GRSP), trace element, and PAH concentrations. Sequencing revealed PAH-degrading fungi. Oil well soils had more PAH-degrading fungi than controls. Next-generation sequencing detected the fungus community and examined its crude oil-contaminated soil bioremediation potential.

Next-generation DNA-metagenomic sequencing was utilised to explore how forest-to-pasture conversion affects soil N-cycle bacteria populations and activity (Pedrinho et al., 2020). N-cycling marker genes assessed nitrogen fixation, denitrification, and other N-related microbes. Aluminum and nitrate greatly influence community structure and 12 of 21 microbial phyla. Forest-to-pasture conversion enhanced nitrogen-fixing *Bacteroidetes, Chloroflexi*, and *Firmicutes*. Grassland abandonment and secondary forest restoration enhanced proteobacteria and denitrification genes. This study's multi-analytical method showed that the secondary forest was robust, suggesting that N-related microbial populations and their potential roles may return, which may effect future ecological restoration efforts.

Next-generation 16S rRNA gene sequencing analysed South African post-coal mining reclamation soil bacterial populations (Ezeokoli et al., 2020). Sequencing data contrasted bacterial population structures in reclaimed and neighbouring unmined soils. CLPP assessed bacterial community function. In reclaimed soils, location and soil history influenced bacterial communities. Reclamation and unmined soils predicted similar bacterial community functions, indicating redundancy.

Next-generation sequencing of the 16S rRNA gene was used to characterise the soil microbial community to monitor deltamethrin degradation on cabbages and nearby soils (Bragança et al., 2019). Monitoring cabbage growth and soil microbial populations determined the pesticide's environmental effect. Deltamethrin and 3-phenoxybenzoic acid conversions impact microbial community composition during 30 days of pesticide application (3-PBA). Deltamethrin was not discovered in soil or cabbage after 180 days, despite its environmental effect. Deltamethrin spraying may disrupt soil microbial population, although spontaneous biodegradation may detoxify pesticide-contaminated soil. The evaluation of soil microbial communities has greatly benefited by NGS, especially in determining the effect of DNA separation techniques on microbial composition. NGS enabled the identification of certain microbial species, including Micrarchaea and Armatimonadetes, and enabled a comparison examination between two investigators using disparate DNA extraction kits (Soliman et al., 2017). Significant variations in genomic DNA yields were also observed. This underlines the need of standard operating procedures to guarantee reliable estimates of microbial diversity in soil ecosystems and emphasises the relevance of NGS in clarifying possible biases imposed by various handling approaches.

### 3.2 Commercial second generation sequencing platforms

#### 3.2.1 Illumina technology

**Fig. 1:**
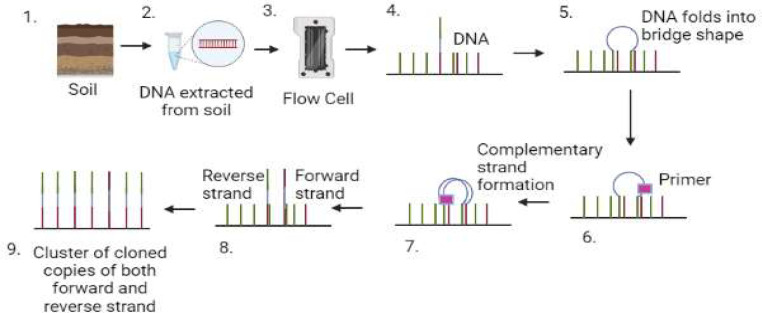
Building Bridges with DNA: Complementary sections creating sturdy bonds through bridge amplification, resulting in a library of clones representing both forward and reverse strands.

The Illumina technology, released in 2006, generates more data with each run in a more economical way. It uses the sequence-by-synthesis method, in which: (1) a flow cell contains a connected oligo field rather than a chip with separate microwells filled with beads; and (2) dye terminators are used instead of pyrosequencing, which resembles Sanger sequencing. Clusters are synthesized by amplifying small DNA segments bound to a glass slide or microwell. The amount of DNA used to load the flow cell needs to be optimized to avoid overloading. Fluorescence-labeled nucleotides integrated in the clustered fragment's complementary DNA sequence are fed through the flow cell. Nucleotide incorporation generates fluorescence, which is detected to determine DNA sequence. The method consists of only three basic steps: amplification, sequencing, and analysis. The process begins off with isolated DNA. Once the DNA has been fragmented, adapters are added. These contain segments that will be used as anchors during amplification, sequencing, and analysis. A flow cell is used to sequence the changed DNA and then amplify it. To keep the fragments from getting too crowded, the flow cell's nanowells are pre-loaded with oligonucleotides that act as anchors for the adapters. Cluster generation starts when the fragments have joined. Using bridge amplification PCR, approximately 1000 copies of each DNA fragment are produced in this step (Fig. 1). The nucleotides are then cleaned and the primers are reapplied once the chip has been modified. The DNA polymerase can only insert one nucleotide at a time into the DNA fragment because of the reversible fluorescent blocker present in these nucleotides. After each synthesis iteration, a photograph of the chip is taken. A computer can record which base was added to each spot on the chip by detecting the fluorescent tag's wavelength. Molecules that were not incorporated are removed after each iteration. The 3′ fluorescent terminus blocking group is then chemically removed. Hundreds of locations throughout the genome are simultaneously sequenced using this method. The procedure is repeated until the entire DNA molecule is sequenced (eg. 2 × 250 bp). Short reads can also be created using this technique. The run time for Illumina HiSeq 2000 has been decreased from 8–11 days to 1-6 days by upgrading to HiSeq 2500 (Nkongolo et al., 2020). The short-run sequencing mode extends the maximum read length from 100 to 150 bp. HighSEqXten, a tailored version of the HiSeq2500, was introduced by Illumina in 2014 (Gruber-Vodicka et al., 2020). Owing to the expensive nature of this device ($1 million per unit), it is anticipated to be unappealing for soil microbiological research. MiniSeq is a compact desktop platform that was introduced in 2011, with an FDA-approved version released in 2013 (Panneerselvam et al., 2020). While it is faster, it produces a lower output. Amplicons are now routinely sequenced using HiSeq and MiSeq (http://www.illumina.com/miseq). HiSeq2000 generates approximately 50 Gb/day. It generates 1.6 billion observations with a 100-base paired end during a 10.8-day run. The MiSeq generates 1.5 Gb/day from 5 million 150-base paired end reads and is designed for single-day analyses. The sequenced parts of the amplicons from these two methods, as well as the biological results they provide, are identical. Hence, the HiSeq2000’s high-throughput microbial community sequencing protocol may be effectively adapted to the MiSeq system, reducing costs and improving the efficiency of the process. The Illumina amplicon sequencing technology, including HiSeq and MiSeq, has become well known as the NGS technology (Sanz and Köchling, 2019; Singh et al., 2022; Edge et al., 2020). In genomics laboratories, Illumina ranks as the top sequencing technique. It has the lowest cost per base and the best throughput while delivering relatively brief readings of up to 300 bp in length. Most applications for additional research can use the Illumina output (Ungelenk et al., 2021). PCR amplicon libraries were generated from three soil sample PCR amplification results to eliminate early round PCR errors (Xu et al., 2017). The purified amplicons were pooled in equimolar quantities and paired-end sequenced (2 × 150) using an Illumina MiSeq platform using standard techniques. After sequencing, quality filtering, resampling, and taxonomic assignment, rarefaction curves, diversity indices, beta-diversity estimates, PCoA, and Venn diagrams identified community differences. Redundancy study analysed samples, environmental conditions, and phylum frequencies. PCR amplicon libraries and Illumina sequencing produced 1,465,650 high-quality tag sequences from 30 soil samples. The four sample sites shared 30% of 7,475 OTUs. Bacterial richness and diversity varied by sample region but not mining or control. PCoA showed considerable clustering within each site and difference between sites, with MY and KM samples being most similar. Illumina's paired-end sequencing instrument's maximum read length varies. Each high-quality paired-end read yielded around 62 nucleotides (nt) sequences from the V6 region of the 16S rRNA gene, accounting for 30.7% of the information in two 101 nt reads (Eren et al., 2013). Shotgun metagenomic sequencing of peat samples on Novaseq was processed using the MATAFILER pipeline53 (Bahram et al., 2022). Quality filtering removed reads less than 70% of the maximum predicted read length (150 bp), with an observed or estimated cumulative error >2 or >2.5 with a probability of 0.01, or >1 ambiguous location. If base quality went below 20 in a 15-base window at the 3’ end or cumulative error exceeded 2, sdm (version 1.46) 43 trimmed reads. All 196 samples have enough readings for statistical analysis.

Illumina Miseq investigated paddy soil arbuscular mycrorrhizal fungal (AMF) population after long-term CO_2_ exposure (eCO_2_) (Panneerselvam et al., 2020). Illumina Miseq high-throughput sequencing of the 18S rRNA small subunit (SSU) gene identified and categorised AMF. SSU gene amplification with AML1/AML2 primers and MaarjAM VT database matching revealed OTU changes in 23 AMF species. AMF community changes were found by Illumina Miseq under ambient (400 ± 10 μmol mol^−1^) and eCO_2_ (550 ± 20) conditions. Long-term eCO_2_ reduced *Glomus* and *Claroideoglomus*. PCO visualised AMF community changes and clustered comparable OTUs, whereas Illumina Miseq showed eCO_2_-repressed or promoted OTUs. High-throughput sequencing by Illumina Miseq revealed AMF community changes, including eCO2-repressed or stimulated OTUs. AMF population was affected by eCO_2_, according to Illumina Miseq.

Illumina-based high-throughput sequencing examined nanozeolite, nanochitosan, and Bacillus sp. on maize rhizosphere microorganisms in the field. Field microbial communities were tested with agiriusable nanocompounds. After 60 days, Illumina sequencing examined the experimental maize rhizosphere's bacteria, soil health indicator enzyme activity, and microbial diversity. Treatment soil boosts OTUs and bacteria (OTUs). Bioinoculant and nanocompounds enhanced FDA, dehydrogenase, and alkaline phosphatase. Actinobacteria, Bacteroidetes, Acidobacteria, and Chloroflexi dominated soil samples. Bacillus sp. and nanocompounds impacted rhizospheric microbial composition, diversity, and richness. Finally, Illumina sequencing allowed field-based nanocompound and bioinoculant impacts on maize rhizosphere microorganisms. Nanocompounds and bioinoculants enhance soil and rhizospheric microorganisms. Illumina sequencing can assess agricultural nanocompounds' long-term soil microbial impacts.

Illumina Miseq-based 16S rRNA amplicon sequencing evaluated two *in situ* mining sites, one overburden soil, and a forest soil near the Odisha Sukinda chromite mines (Pradhan et al., 2020). Bioremediation microorganisms tolerated metals. Illumina Miseq high-throughput sequencing of 16S rRNA amplicons revealed research site bacterial diversity and community structure. Almost 20,000 OTUs from seven bacterial phyla—Actinobacteria, Proteobacteria, Firmicutes, Acidobacteria, Chloriflexi, Bacteroidetes, and unclassified—were collected. Medium-polluted overburden has the most bacterial OTUs. Heavy metal pollution affected bacterial community diversity and organisation *in situ*. Metal-tolerant Actinobacteria, Proteobacteria, and Acidobacteria moved to controls. Shigella, Bacteroides, Propionibacterium acnes, Pantoea, Aciditerrimonas, Reyranella, Alphaproteobacterium, and Burkholderiaceae prevailed. Illumina Miseq discovered metal-tolerant microorganisms in bioremediation. Microorganisms bioremediated Sukinda chromite mine. Heavy metals changed soil bacterial populations according to Illumina Miseq sequencing.

Populations of arid fungi were characterised using Illumina sequencing (Hamm et al., 2020). In an arid Utah grassland, biocrust and rhizosphere soils containing bait produced keratinophilic fungus. The cultured fungus was identified by the ITS and LSU rRNA sequences. Illumina sequencing revealed the fundamental seasonal fluctuations and habitat preference of keratinophiles. The sequencing of the DNA of the fungal communities revealed species, abundance, and variation. According to Illumina sequencing, over 70% of the keratinophilic fungi in the samples were Alternaria. Dermatophytes, which are fungi that can cause skin diseases, were scarce. Seasons and ecosystems had an impact on fungi. Illumina sequencing revealed the species, abundance, seasonality, and habitat preferences of arid fungi. In this arid environment, Illumina data and culture-dependent approaches revealed a specialised population and low dermatophyte abundance.

#### 3.2.2 Ion torrent technology

**Fig. 2:**
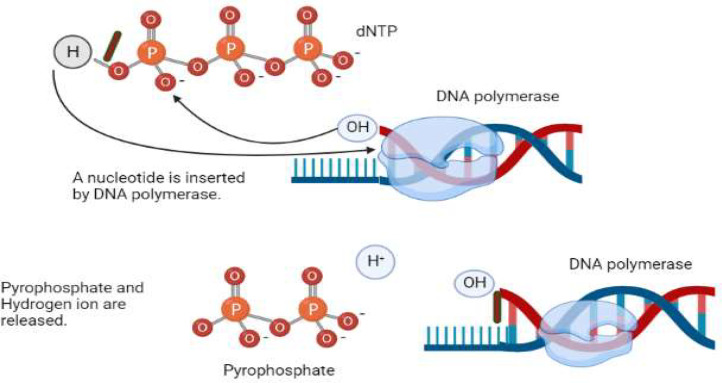
Pyrophosphate and a hydrogen ion are released when a deoxyribonucleotide triphosphate is incorporated into the developing DNA strand.

The principle of the ion torrent technology (Fig. 2) is similar to that of pyrosequencing in that the DNA is sequenced by tracking the addition of each nucleotide. When a new nucleotide in the form of dNTP is added to the developing strand of DNA, a covalent bond is formed and a pyrophosphate and a proton are released. A covalent bond is formed only when the complementary nucleotide is added. Ion semiconductor sequencing benefits from the release of the proton. Nucleotides A, T, G, and C are sequentially injected into the reaction mixture. Each microwell within the semiconductor chip is made up of multiple copies of one template and a DNA polymerase. The complementary pairing of A-T and C-G leads to the addition of the nucleotides with the help of DNA polymerase. If the wrong nucleotide is added due to an error, that nucleotide is removed before the next cycle starts. The release of a proton at every nucleotide insertion decreases the pH of the reaction mixture, which is monitored by an ion-sensitive field-effect transistor. DNA fragments are bonded to beads in the ion torrent method, and each microwell is filled with beads. Each of the four nucleotides passes through the wells and joins a complementary strand, resulting in the release of an H^+^ ion and a voltage change that can be detected (Meera Krishna et al., 2019). This procedure is repeated several times. Ion torrent technology generates reads of up to 400 bp in length and can complete a run faster than other platforms. Unfortunately, this method is not as widely used as Illumina technology, probably because of its high polymeric error rate. While isothermic PCR is used to create templates in Illumina sequencing, emulsion PCR is used in ion torrent sequencing. Ion torrent enables extensive, cost effective sequencing of fungal and bacterial populations. However, earlier iterations had issues with reading length and sequence quality (Altermann et al., 2022). Research on microbial communities has not frequently employed ion torrent sequencing because compared to pyrosequencing, ion torrent sequencing employing 100 or 200 bases results in sequence lengths of <250 bp with worse taxonomic resolution (Nkongolo et al., 2020). The results of soil bacterial community profiling showed that when used for semiconductor sequencing, the ion torrent technology exhibited greater rates of inaccuracy and a propensity for early gene termination compared to the Illumina technology (de Melo Pereira et al., 2022). Primer lengths of up to 410 bp may now be produced for 16S rRNA, owing to the availability of 400 nucleotides for ion torrent sequencing, which is similar to the 454 GS Junior pyrosequencing. Currently, the most widely used version of this technology for studying soil microbial communities is the Ion Torrent Personal Genome Machine (PGM). PGM measures the pH change caused by the release of the hydrogen ion during nucleotide integration, as opposed to using expensive light detection equipment.

Ion Torrent assessed soil and lab-grown efficient bacteria EM16 consortium (Carabeo et al., 2022). Hydrogen ions real-time sequence DNA. PGM sequenced EM and soil DNA fragments. Ion Torrent, microbiological, and molecular methods evaluated microbial populations. Enzyme-produced DNA fragments recognised microbes. Ion Torrent found cultivable and non-cultivable microbes. The EM16 consortium and soil isolates from agricultural residues (sugarcane crop and bamboo field) showed potential for bioremediation and sustainable crop production due to their diverse metabolic pathways and symbiotic interactive potential for biodegradation of lignocellulosic-resilient compounds. IonTorrent measured soil and EM microbes. This approach showed microbial community richness, dynamics, taxonomic identification, bioremediation, and sustainable agriculture.

Ion Torrent sequencing evaluated organic cowpea (*Vigna unguiculata*) and melon (*Cucumis melo*) intercropping and monocropping bacterial communities (Cuartero et al., 2022). Research shows intercropping alters soil nutrients, physicochemical qualities, enzyme activity, and microorganisms. Dneasy Power Soil Kit extracted soil DNA, while Qubit 3.0 Fluorometer and NanoDrop 2000 fluorospectrometer analysed DNA quality and quantity. Ion TorrentTM PGM System sequenced bacterial 16S hypervariable regions. Proteobacteria, Actinobacteria, Acidobacteria, Firmicutes, Gemmatimonadetes, Planctomycetes, Chloroflexi, Bacteroidetes, and Nitrospirae dominated soils utilising Ion Torrent sequencing. Cowpea suppresses melons’ microbiota. Intercropping increased melon monocrop nitrogen, accessible phosphorus, total organic carbon, acid phosphatase, and β-glucosidase. *Pseudomonas, Bacillus, Streptomyces*, and *Sphingomonas* were discovered.

Ion Torrent sequencing evaluated soil bacterial populations before and after zerovalent iron grit, natural zeolite, and Divergan® treatment (Kaplan et al., 2019). Bacterial community profiling using copper and cadmium-resistant 16S rRNA genes (copA and czcA gene). Ion Torrent sequencing showed soil samples’ microbial community structure and function better than culture-based approaches. DNA sequencing identifies hydrogen ions quickly, accurately, and cheaply. Following remediation, soil 16S rRNA gene sequencing revealed microorganisms. Treatment improved *Kribbella, Glycomyces, Inquilinus, Nocardioides*, and *Lysobacter*. Remediated soils lowered efflux-mediated Cd/Zn and Cu metal resistance bacterial groups. Sequenced Xanthomonadaceae has the most copA and czcA genes. Ion Torrent sequencing revealed soil microbial community structure and function before and after remediation and the long-term effectiveness of the three trace metal additions.

### 3.3 Third and fourth generation sequencing platforms

Newer, more effective sequencing methods, the third and fourth generations, were developed in response to the limitations of the previous generation's methods (Bleidorn, 2016). Third and fourth generation sequencing methods, also known as long read technologies, represent a radical departure from earlier sequencing methods since they are designed to sequence extremely long DNA and RNA molecules. The third generation can generate read lengths for >10 kb and does not need library preparation of DNA template to be sequenced, i.e. it can execute sequencing operations directly from the native DNA, eliminating the manipulation of template sequencing and decreasing the sequencing error rates. Pacific Biosciences (PacBio) and Oxford Nanopore Technology are two pioneers in this field (ONT). These two sequencing methods create enormous amounts of data, but they operate on fundamentally distinct tenets. Analysis of molecules using longer read lengths from sequencing reduces time and money without sacrificing accuracy throughout the genome (Midha et al., 2019).

#### 3.3.1 Pacific Biosciences (PacBio) or Single molecule, real time (SMRT)

**Fig. 3:**
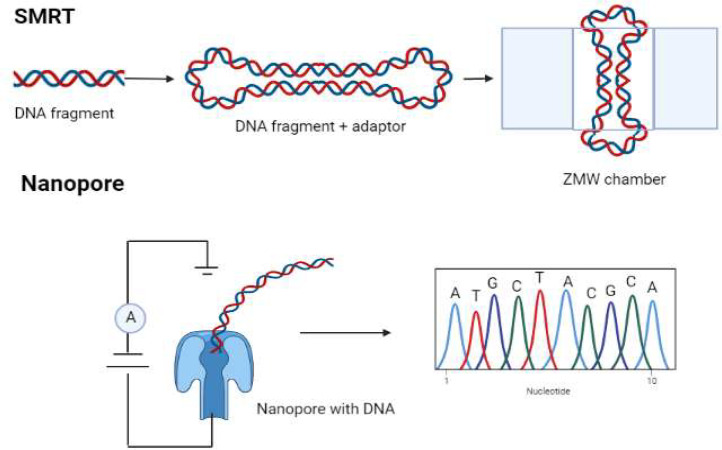
Unaltered DNA can be used to detect methylation via Oxford Nanopore and Pacific Biosciences (PacBio) single-molecule real time (SMRT) technologies. ZMW, zero-mode waveguide.

The method of single-molecule real-time (SMRT) sequencing is a parallel approach of sequencing a single DNA molecule. The zero-mode waveguide (ZMW) used in SMRT consists of a silicon substrate with a circular hole drilled into it and an aluminium cladding layer. The holes are around 70 nm in radius and 100 nm in depth. A template, a sequence primer, and a DNA polymerase make up the polymeric unit of every ZMW (Baum, 2021). DNA polymerase, which is attached to the ZMW's underside, acts as a guide in the formation of one DNA strand. One of the four fluorescent dyes is linked to each of the four DNA bases. The DNA polymerase removes the fluorescent tag as it integrates a nucleotide, and the tag's absence from the ZMW chamber results from its diffusion outside the chamber. The insertion of a nucleotide causes a change in the fluorescence of the dye that corresponds to the base, and this change may be measured by a detector. DNA sequencing is a breeze on a device with a lot of ZMWs. DNA polymerase activity and ssDNA transport may be observed in the ZMW's observation chamber. At each stage of DNA synthesis, the fluorescent dye molecule picks up a signal from the phosphorylated nucleotides. The fluorescent dye is linked to the nucleotide's phosphate molecule; when the phosphodiester bond is formed during nucleotide integration to the DNA strand, the dye is released. As soon as the dye is liberated from the fluorescent molecule, the fluorescence signals cease since the dye has diffused out of the detecting volume of ZMW (Fig. 3). In order to make libraries, adaptors are ligated to hairpin loop structures. Because of how light behaves when it passes through a small opening, the quality of the optical field inside the chamber degrades exponentially. In a well-lit ZMW, the volume of space available for viewing is on the order of 20 zeptoliters (20 1021 litres). With the addition of a single nucleotide, DNA polymerase activity may be readily seen in this volume. For SMRT, Helicos delivered the first commercial solution based on fluorescence detection and sequencing by synthesis (Ardui et al., 2018). PacBio is based on cutting-edge SMRT sequencing technology. In the future, systems will be built with inexpensive and straightforward nanopore technology. Genome reconstruction from metagenomic data is anticipated to benefit from the use of long reads, which will allow researchers to investigate genomic reorganisation and metaregulomes in various microbial communities (Bharti et al., 2021). There are, however, certain downsides to utilizing this cutting-edge technology. With an average read length of 15 kb, the PacBio RSII produces only 50,000 reads per SMART cell, which is comparable to 1 GB in each SMRT cell (Nkongolo and Narendrula-Kotha, 2020). When compared to parallel sequencers like MiSeq (Illumina), which produce over 15 GB every run, this is a significant reduction. Therefore, it is possible that communities home to a great variety of species will defy PacBio RS II's ability to characterise them. PacBio's long-read technology has low accuracy and high base error rates because of the fluorescent tags attached to the terminal phosphates of the phospholinked nucleotides that participate in polymerization. Light sent out by the stimulated, excited tag is picked up by an optical detector. At the conclusion, the fluorescent label is removed, and a polymerization complex ready to attach to strands is made (Ren, 2018). Although a considerable quantity of DNA sequences is needed as input for PacBio sequencing technologies, the resulting reads can be up to 10-15 kb in length, with some reads reaching 50,000 bp. While the 15% raw error rate in PacBio sequencing is somewhat high, it is correctable and may be used to get extremely accurate findings (Wei et al., 2018).

ONT incorporates single-molecule sequencing. Nanopore sequencing makes use of the fact that single-stranded DNA (ssDNA) undergoes a change in electric charge as it travels through a protein channel (a nanopore). DNA polymerase contains an enzyme that lets single-stranded DNA (ssDNA) go through a nanopore (Brinkerhoff et al., 2021). The order of the nucleotides on the DNA scaffold is identified by a change in electric current caused by the passage of DNA bases through the nanopore. The MinION sequencing equipment from ONT are the size of a palm but produce lengthy readings in real time. Upon its release, MinION generated read lengths of around 6-8 kb; however, a lab practice may boost the MinION readings and yield many reads of >100 kb. Adaptive error rates are challenging in ONT technologies, just as they are in PacBio (Tyler et al., 2018).

### 3.4 Some ancillary techniques for metagenomics studies

#### 3.4.1 Metagenomics and metabarcoding

Soil microbiologists have made significant use of metagenomics and metabarcoding, two highly effective methods for examining the structure and function of soil microbe populations. Rapid and high-throughput analysis of vast volumes of genetic data is made possible by these methods, allowing for a complete portrait of soil microbial communities to be compiled. In soil metagenomics, all of the microbial DNA is sequenced to learn about the many kinds of bacteria present and how they work together. Instead, metabarcoding is a focused method that employs gene markers to isolate and count just the types of microorganisms that are present in a given sample. Our knowledge of soil microbial communities, their function in soil processes, and their susceptibility to external influences has been bolstered by the use of metagenomics and metabarcoding in soil microbiology. Soil microbial communities, for instance, have been the focus of metagenomics research on the effects of land use change and soil management methods (Fierer et al. 2012; Liang et al. 2019). Fungal community diversity and composition have been quantified via metabarcoding, shedding light on the important role fungi play in soil processes (Choat et al. 2012; Hounjet et al. 2017). A comparison amog different complementary appraochs is listed in Table 2.

**Table 2:** A comparative study of different metagenomic approaches for the investigation of soil microbes

Metagenomic Technique	Applications	Advantages	Disadvantages (Limitations)	Operative Conditions	Major Factors Impacting	References
Illumina	Analysis of microbial diversityHigh-throughput sequencing and functional metagenomics	Budget-friendly- Complex genome	assembly is hampered by short read lengths.-	Obtaining DNA from soil samples- Library setup (PCR amplification, indexing, etc.)- Illumina platform sequencing-	Depth of the sequence Reading length- Quality of library preparation- Bioinformatics analysis techniques and pipelines	Chaudhary et al., (2021)
Single Molecule Real Time (SMRT)	High quality de novo construction	Long read length	More errors per unit of data than short-read technologies	Obtaining DNA from soil samples- Library setup (e.g., SMRTbell setup) PacBio SMRT sequencing systems for sequence analysis.	Accuracy and error rate of sequencing- Library-caliber- Tools for long-read data analysis	Hon et al., (2020)
Microarray	Gene expression analysis	Concurrent high-throughput investigation of many targets	Limited coverage in comparison to approaches based on sequencing	Soil sample extraction of DNA or RNA. The hybridization and scanning of microarray chips	The design and effectiveness of microarray probes- Techniques for normalising and analysing data.	Kumar et al., (2021)
Clone libraries	The discovery of genes- Characterization of the function	Allows for the isolation of certain clones for further research.	Inadequate representation and coverage of microbial diversity	Obtaining DNA from soil samples. Cloning DNA fragments into the appropriate vectors- Sanger clone-by-clone sequencing	The size and complexity of the library- Target gene screening and selection techniques- Coverage and quality of the sequencing	Santana-Pereira et al., (2020)
SoLID (Sequencing by oligonucleotide ligation and detection)	De novo Resequencing	Long read distances	Lower throughput compared to other sequencing systems and limited availability	Obtaining DNA from soil samples- Library construction (for instance, oligonucleotide ligation)- Sequencing on platforms for SoLID	Readability and precision- The standard and complexity of libraries- Chemistry and instrument performance	Sehgal and Chaturvedi (2022)
GeoChip	Functional gene profiling	- Facilitates the high-throughput identification of functional genes	Limited coverage in comparison to approaches based on sequencing	Obtaining DNA from soil samples- Microarray hybridization using Geochip using retrieved DNA	Microarray probe design and coverage- Techniques for optimization and analysing data.	Nkongolo et al.,(2020)
DNA stable isotope probing	Determination of populations of active microorganism	Allows for the monitoring of the assimilation of stable isotopes by microbes	Requires specialised methods for isotope labelling and detection-	Stable isotope incorporation into soil samples and DNA extraction from labelled samples- DNA labelling and sequencing	Techniques for labelling isotopes- DNA extraction bias and effectiveness- Depth and coverage of sequencing	Alcolombri et al., (2022)
Mobile metagenomics	Investigation of Mobile Genetic Elements (MGEs)	Makes it possible to locate and characterise mobile genetic components in intricate soil microbial communities	Difficulties in putting together mobile genetic elements and identifying their hosts	Obtaining DNA from soil samples- The creation of libraries using transposons, for example.- NGS platform sequencing	Methods for preparing libraries for mobile components- Sequencing breadth and depth- Bioinformatics tools for mobile element analysis	Brito (2021)
Plasmid metagenomics	Research into plasmid dynamics and diversity	Makes it possible to find and analyse plasmids in soil microbial populations.	Difficulties in plasmid assembly and identifying their hosts	Obtaining DNA from soil samples- Plasmid DNA enrichment. The creation of libraries using transposons, for example.- NGS platform sequencing	Techniques for plasmid DNA enrichment- Sequencing breadth and depth- Plasmid analysis and identification bioinformatics tools	Wang et al., (2021)
Shot gun metagenomics	An extensive examination of the composition and activities of microbial communities.	Enables thorough and objective study of the functional capacity of the microbial communities in soil.	Difficulties in integrating complicated metagenomic data and putting unknown sequences to use	Obtaining DNA from soil samples- The creation of random fragment libraries, for example.- NGS platform sequencing	Sequencing coverage and depth- Assembly techniques and algorithms- Tools for analytical and functional annotation	Lu et al., (2023)

In order to fully understand the genetic diversity of soil microorganisms and, in particular, to examine the makeup of communities using barcode marker gene sequences, metabarcoding is essential to research. These molecular biology techniques, in conjunction with metagenomics, provide direct insights into the great genetic diversity of the "uncultivated majority" of soil microbes (Semenov 2021). The research study by Procopio et al., (2019) used a bacterial 16S rRNA V4 region sequencing method, known as metabarcoding, to investigate the succession of soil microbial communities linked to the decomposition of pig corpses at different post-mortem intervals (PMIs). A drop in Acidobacteria, a rise in Proteobacteria, Firmicutes, and Bacteroidetes at certain PMIs, and the preservation of some mammal-derived taxa, such as Bacteroides spp., even after six months PMI were among the unique patterns in microbial taxa that the metabarcoding data indicated. The research showed that metabarcoding may be useful for determining post-mortem periods, especially when dealing with cases of severely skeletonized remains or secret burials where the corpses are moved. Joos et al., (2020) demonstrated that in order to compare Operational Taxonomical Units (OTUs) and Amplicon Sequence Variants (ASVs) to analyse the richness, structure, and complexity of microbial communities, metabarcoding was essential. Changes that affect the biological interpretation of data were found when metabarcoding analysis switched from using OTUs to ASVs, especially in habitats with high microbial diversity. The work highlighted the significance of thorough research and suggested that, in order to draw valid biological findings from metabarcoding investigations of microbial communities, comprehensive sequencing, suitable filtering techniques, and the application of family-level clustering should be used.

#### 3.4.2 Clone libraries

Metagenomics studies soil microbial diversity through clone libraries. Microorganisms recycle nutrients, grow plants, and bioremediate soil. Clone libraries may show these microbes' genetic diversity. PCR primers targeting conserved genes like the 16S rRNA gene amplify ambient DNA to produce clone libraries. PCR converts *Escherichia coli* with plasmids. Environmental DNA-cloned bacteria thrive. DNA sequencing checks clone library diversity. Sequencing cloned DNA segments shows microbial community taxonomic and functional diversity. Soil metagenomics examines bacteria, fungus, and archaea using clone libraries. Diversifying soil fungus using PCR primers targets the fungal internal transcribed spacer (ITS). Bioinformatics can assess microbial taxonomic diversity and abundance following clone library sequencing. Phylogenetic trees and statistics may indicate organism links. Researchers may track community changes using clone libraries. Clone libraries include new microbial species and soil microbial community activities including pollution degradation and plant growth. Metagenomics clone libraries examine soil ecosystem microbial diversity. Using clone libraries, soil bacteria's complex ecological processes may be displayed.

**Fig. 4:**
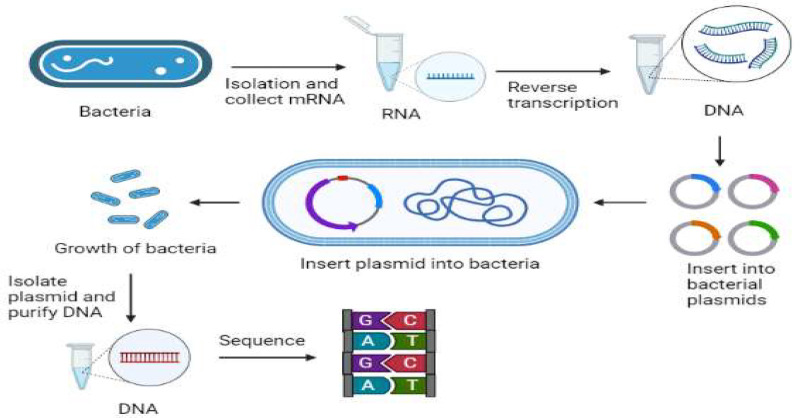
Production of cDNA libraries.

Reverse transcriptase is used to convert mature mRNA extracted from eukaryotic cells into cDNA. An extended sequence of adenine nucleotides, known as the poly-(A) tail, serves as the primer binding site for reverse transcription and separates mRNA, tRNA, and rRNA in eukaryotes. The poly-A tail is not encoded in all transcripts, such as those encoding histones, which poses a problem. The first step in creating cDNA libraries is to isolate the mRNA template. The integrity of the separated mRNA should be considered because mRNA only contains exons, ensuring that the encoded protein can still be generated. The size of the isolated mRNA should be between 500 bp and 8 kb. Several techniques for RNA purification, including triazole extraction and column purification, are available. Distinct mRNA characteristics, such as the presence of a poly-A tail, can be exploited by using oligomeric dT nucleotide-coated resins in column purification, in which only mRNA sequences with a poly-A tail can bind. After the mRNA binds to the column, it is eluted. An oligo-dT primer, which is a brief strand of deoxythymidine nucleotides, is attached to the poly-A tail of the RNA after the mRNA has been purified. The reverse transcriptase enzyme requires a primer to initiate DNA synthesis. As a result, RNA–DNA hybrids, wherein a single complementary DNA strand is coupled with an mRNA strand, are produced . The RNAse H enzyme eliminates the mRNA by cleaving its backbone and producing free 3′-OH groups, which are crucial for the replacement of mRNA with DNA. The cleaved RNA serves as a primer for DNA polymerase I to begin replacing RNA nucleotides with DNA. DNA polymerase I is provided by the ssDNA itself, which coils around itself to form a hairpin loop at the 3′ end. The 3′-OH end is extended by the polymerase; eventually, the 3′ end loop is opened by the scissor activity of the S1 nuclease. The sequences are then cloned into bacterial plasmids using DNA ligase and restriction endonucleases. The bacteria for plasmid insertion are usually chosen through antibiotic selection. Bacterial stocks are prepared, allowing further growth and sequencing to build the cDNA library (Fig. 4).

#### 3.4.3 GEOChip

**Fig. 5:**
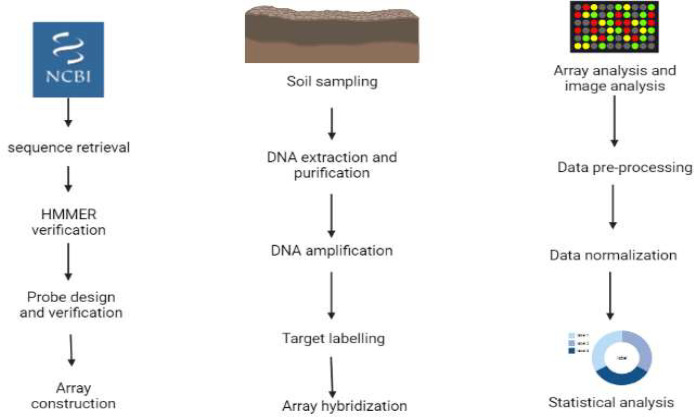
GeoChip development, setting up the target, analysis of the GeoChip data, and GeoChip applications. GeoChips are used to analyze microbial communities from various settings.

A functional gene array technique called GeoChip permits the analysis of thousands of genes all at once. The human microbiome isn't the only thing scientists have used it to investigate; polluted sites, harsh environments, bioleaching systems, and bioreactors are all places where microbial populations have been studied to learn more about their genetic variability, organisational structure, and interactions (Srivastava et al., 2019). A GeoChip development and data analysis pipeline can aid in the following processes: selecting a target gene, retrieving and verifying its sequence, designing an oligonucleotide probe, validating the probe, building an array, and implementing regular updates (Fig. 5). This approach has been proven to be an effective tool for characterising microbial populations in a variety of settings. High-throughput metagenomics is a useful method for studying the effect of microbial communities on ecosystem services, including their diversity, composition, and function. The majority of efforts put into developing probes have been directed towards genes involved in stress response, organic pollutant degradation, antibiotic resistance, secondary metabolism, virulence factors, geochemical cycles, metal homeostasis, viruses, protists, and fungal specificity. NimbleGen created GeoChip and offers it commercially or independently. All oligonucleotides used in the internal array are at a concentration of 100 pmolL^−1^, and all probes used are commercially produced. Every sub-grid has a total of nine control probes, with three of each type (positive, negative, and neutral) present. All of the oligonucleotide controls and probes were spotted onto Corning UltraGAPS slides using an array spotter. To the tune of 2.1 million probes per slide, NimbleGen's micromirror array synthesis is a game changer. GeoChip 5.0 is the newest and most reliable release. GeoChip 5.0, built on the Agilent platform, contains 167,044 individual probes that collectively cover 395,894 CDS from more than 1,500 functional gene families. It has been shown through both experimental and computational testing that GeoChip 5 has excellent sensitivity, specificity, and quantitative performance. Over 3,70,000 sequences from 1,500 gene families are covered by its 1,60,000 probes (Nkongolo and Narendrula-Kotha, 2020). This microarray allows for the most in-depth functional examination of microbial populations to date, which is crucial for ecology, environmental research, biogeochemistry, and human health (Shi et al., 2019). Despite these benefits, early FGAs had limits in probe coverage, specificity, quantitative capabilities, nucleic acid purity, and the ability to detect microbial community activity. Moreover, there are already so many functional gene sequences in databases that the coverage of GeoChip probes is inadequate. Prior research using shotgun metagenomics sequencing has uncovered far lower levels of genetic diversity than GeoChip discovered on average for the genes under analysis. When comparing populations, GeoChip was able to detect more variant genes than the shotgun approach (Shi et al., 2019).

#### 3.4.4 DNA stable isotope probing

**Fig. 6:**
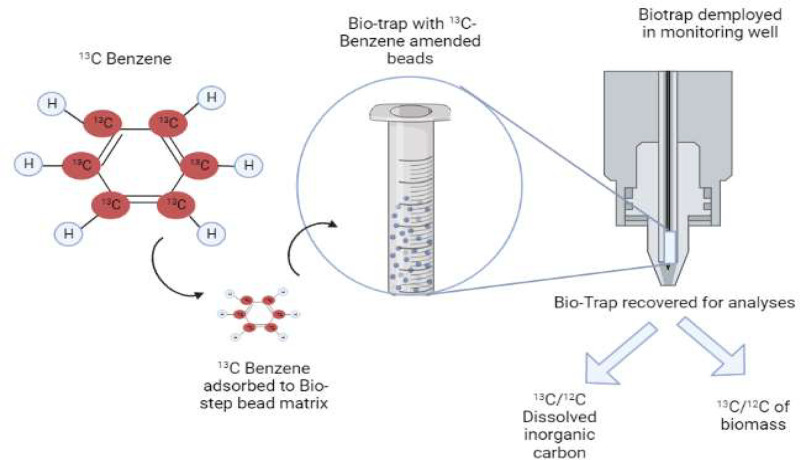
Stable isotope probing (SIP) in use: loading, deployment, and recovery of ^13^C-labeled benzene from a Bio-Trap® passive sampler.

A potent technique for finding and characterizing microorganisms *in situ* and connecting microbial metabolic activity to taxonomic identification is DNA stable isotope probing (DNA-SIP) (Lerner et al., 2020). In general, all SIP methods involve isotopically tagged pollutants to detect, measure, and identify the microorganisms involved in biodegradation. SIP has become more popular in recent years for the identification of isotope absorption by microbial species *in situ* (Bhadury and Ghosh, 2022). This method calls for pollutants that have been enhanced artificially with high concentrations of stable isotopes, such as benzene with ^13^C and ^15^N. In a typical SIP study, a passive microbiological sampling tool, such as Bio-Sep® beads inside a Bio-Trap® sampler, is adsorbed to a ^13^C version of the pollutant, such as ^13^C-benzene (Fig. 6). Nomex® and powdered activated carbon (PAC) are combined to create Bio-Sep® beads, a designed composite. The ^13^C-labeled substance is adsorbed by PAC, which also offers a sizable surface area for microbial colonization and expansion. The beads can be heat sterilized before the study with Nomex®. Installation of a passive sampler allows for efficient monitoring. The ^13^C-labeled contamination is exposed to the same microbiological activities as unlabeled contaminants at the site for the deployment duration, typically 30–90 days. A variety of pollutants are required for microbial growth, including petroleum hydrocarbons, as a carbon and energy source. The ^13^C label will therefore be integrated into the microbial biomass or become ^13^CO_2_ in case of biodegradation. The contaminant-degrading bacteria will colonize the Bio-Trap® and use the ^13^C-labeled contaminant as a carbon and energy source for growth. Following deployment, the Bio-Trap® is retrieved for examination using isotope ratio mass spectrometry and gas chromatography to determine the biomass and dissolved inorganic carbon (DIC) ^13^C/^12^C ratios. The ^13^C label will become part of the microbial biomass or mineralize as ^13^CO_2_. It is clear that *in situ* biodegradation took place when ^13^C-enriched biomolecules, such as phospholipids, DNA, or proteins and ^13^C-enriched DIC are found after deployment. In contrast, the ^13^C/^12^C ratio of the microbial biomass and DIC will be comparable to the background values in the absence of biodegradation. As phospholipid fatty acids (PLFA) are an important part of microbial cell membranes, ^13^C-enriched PLFA clearly indicates that ^13^C has been incorporated into biomass. Likewise, mineralization can be indicated by ^13^C-enriched DIC, such as CO_2_. SIP examines the changes in the isotopic composition of proteins, lipids, nucleic acids, and other microorganism-derived macromolecules. This method is also used to evaluate the biodegradation of several pollutants, including polycyclic aromatic hydrocarbons, fuel oxygenates, insecticides, and gasoline components, and to track nutrient fluxes in biogeochemical cycling by microorganisms. More significantly, it has been proven to be a useful tool for determining the metabolic role of the many communities that live in various terrestrial and aquatic habitats.

For instance, this technique, can identify the organisms that actively ingest or photosynthesize fresh photosynthate. Several commercial applications of this techniques for envrironmental research have been introduced owing to its inherent simplicity and adaptability. HTS can be used with DNA-SIP to study intricate background ecosystems (Hatzenpichler et al., 2020). HTS-DNA-SIP has made it possible to determine isotope incorporation patterns in several microbial species (Teng et al., 2021). The fundamental benefit of SIP is the ability to examine different environmental samples, such as water, soil, and sediment, without the need to first identify the organisms involved in the biodegradation of the contaminants. Any contamination can be examined through SIP as long as its isotopically enriched forms are available. However, SIP has certain limitations. Experiments involving DNA-SIP are expensive, technically challenging, and time consuming. Further, the cost and implementation time for various SIP approaches vary depending on the isotopically labeled molecules. SIP results should be extrapolated with caution to actual field situations. Certain circumstances that are needed to obtain a visible SIP signal could be very different from what is actually happening belowground.

#### 3.4.5 Mobile metagenomics

To improve such circumstances, mobile genetic components with genes for antibiotic resistance can be implanted. Hubs for resistance gene synthesis, screening, and horizontal transmission may exist in ecosystems with genetically diverse ambient bacteria, human commensals, and illnesses. Owing to this, these ecosystems are fascinating from both academic and global health perspectives. Resistance genes and mobile genetic elements were abundantly found when metagenomic DNA from antibiotic-contaminated sediments in a river receiving PETL-treated effluent was subjected to shotgun sequencing (Liet al., 2020). Using a functional metagenomics technique, several new resistance genes were identified, including a novel extended spectrum beta-lactamase that can hydrolyze vital drugs like carbapenems and cephalosporins (Marathe et al., 2018). We found substantial evidence indictating that these genes were mobile according to assessments of their surrounding genomic environment, signifying elevated hazards, even though separating mobile from immobile genes is frequently challenging in functional metagenomics.

#### 3.4.6 Plasmid Metagenome

**Fig. 7:**
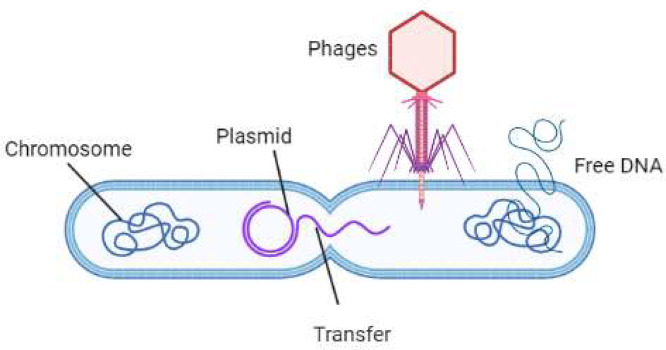
Phage genome integration into a bacterial plasmid.

A detailed investigation of the microbiota and mobile resistome profiles of distinct habitats is required to understand how the environment influences antimicrobial resistance, one of the largest dangers to global public health (Singh et al., 2018). MRGs for resistance to arsenic, copper, zinc, and molybdenum were observed in the majority of the samples (Lu et al., 2022). Except for mobilome genes, which are frequently identified in plasmid sequences, the majority of resistome genes has been found in chromosomal sequences (Fig. 7). All samples had mobile genetic elements related to transposases. The bulk of the data included mobile genes or genes associated with plasmids that conferred resistance to antibiotics, like colistin and carbapenems, until their last days (Cerezales et al., 2020).

## 4 Conclusion and future perspectives

HTS methods coupled with genomic arrays, bioinformatics, expression-based assessments, and standard methods can be used to understand several agriculture factors, including soil quality and conservation, crop yields, and synthetic biological mechanisms, which are in addition to microbial strains and biochemicals that are limited by our inability to isolate most microbes. The review emphasises that targeted metagenomics, makes use of methods like stable-isotope probing and high-throughput sequencing, is crucial for examining particular microbial taxa and genes in intricate ecosystems. In order advance soil microbiome research, the paper highlights the revolutionary influence of many sequencing technologies, such as Illumina, Ion Torrent, Oxford Nanopore Technology (ONT), and Pacific Biosciences (PacBio). GeoChip, clone libraries, metagenomics, and metabarcoding are examples of ancillary tools that enhance our knowledge of soil microbial populations. Despite certain challenges, metagenomics has great promise for improving agricultural practises and environmental management. By revealing the whole range of microbial diversity, roles, and interactions, metagenomics will revolutionise soil microbiology research. The future of soil microbiology research is expected to be shaped by the continuous development of metagenomics. More comprehensive metagenomic sequencing procedures are warranted to understand the diversity of the soil microbiome and provide appropriate information to comprehend the diversity and activity of the soil microbial community. Appropriate sampling, efficient DNA extraction, replication, screening, and sequence analysis along with enhanced open network information management and bioinformatics are necessary for the field of soil metagenomics. The stability of the ecosystem is based on soil quality, which is influenced by soil microorganisms. Soil quality also influences crop yield. However, current knowledge on soil microorganisms is limited. Metagenomic tools could provide not only a library of millions of living organisms but also allow the investigation of the associations between microbes, soil, and crops. Thus, microbial communities in soils could be used to develop better, more resilient crops and novel biomolecules. This “hidden natural wealth” must be conserved. The soil environment harbors important and unexplored variety of resources with various bioactivities and environmental roles.

## Funding

This work was supported by a KAUST Baseline Grant (to Prof. A. S. Rosado) (BAS/1/1096-01-01).

## CRediT authorship contribution statement

**Diksha Garg:** Conceptualization, Methodology, Data curation, Writing – original draft, Writing – review & editing. **Niketan Patel:** Conceptualization, Methodology, Data curation, Writing – original draft, Writing – review & editing. **Anamika Rawat:** Data curation, Writing – original draft, Writing – review & editing. **Alexandre Soares Rosado:** Data curation, Writing – original draft, Supervision, Writing – review & editing.

## Declaration of competing interest

The authors declare that they have no known competing financial interests or personal relationships that could have appeared to influence the work reported in this paper.

## Data Availability

No data was used for the research described in the article.
